# Comparative efficacy of Chinese herbal injections combined with GP regimen chemotherapy for patients with advanced NSCLC: A protocol for systematic review and network meta-analysis

**DOI:** 10.1097/MD.0000000000021041

**Published:** 2020-07-10

**Authors:** Juan Li, Guang-Hui Zhu, Tong-Tong Liu, Bo-Wen Xu, Jie Li

**Affiliations:** a Department of Oncology, Guang’anmen Hospital, China Academy of Chinese Medical Sciences; b School of Graduates, Beijing University of Chinese Medicine, Beijing, China.

**Keywords:** Chinese herb injections, GP, network meta-analysis, NSCLC, protocol

## Abstract

**Background:**

Many research has indicated that some Chinese herb injections (CHIs) might be beneficial in combination with chemotherapy, however, with inconsistent results. Hence, the purpose of this network meta-analysis is to evaluate different CHIs plus cisplatin and gemcitabine (GP) with GP alone in terms of clinical efficacy and safety for treating patients with advanced NSCLC.

**Methods:**

A comprehensive systematic search of clinical randomized controlled trials (RCTs) published in the PubMed, Embase, Web of Science (ISI), Cochrane Central Register of Controlled Trials (CENTRAL), China National Knowledge Infrastructure Database (CNKI), Chinese Scientific Journals Full-Text Database (VIP), Wanfang Database and China Biological Medicine Database (CBM) databases will be conducted to identify eligible studies up to the date of May 2020. The primary outcome measures objective response rate and adverse reactions (nausea and vomiting, leukopenia). The secondary outcome measures median survival time (MST), disease control rate, and quality of life. The methodological qualities, including the risk of bias, will be evaluated using the Cochrane risk of bias assessment tool, while confidence in the cumulative evidence will be evaluated using the Grading of Recommendations Assessment, Development and Evaluation (GRADE) approach. The network meta-analysis will be performed using WinBUGS 14 and Stata 15.1 software.

**Results:**

Based on the current evidence, the potential rank of the efficacy and safety of CHIs plus GP chemotherapy for advanced NSCLC will be assessed, and a prioritization regimen will be summarized.

**Conclusion:**

Evidence from this systematic review could be useful for patients, clinical practitioners, and guideline-makers to select an optimum proposal of CHIs plus GP for advanced NSCLC.

**Ethics and dissemination:**

It is not necessary for ethical approval because it is based on published studies. The protocol will be disseminated in a peer-reviewed journal or presented at a topic-related conference.

**PROSPERO registration number:**

CRD42020167142

## 1 Introduction

Lung cancer, whose morbidity rate is continuing to rise, is one of the malignant tumors with the highest morbidity and mortality in the world. Nearly 85% of lung cancers are nonsmall cell lung cancer (NSCLC).^[1]^ Surgery is the first choice for early NSCLC, but nearly 75% of patients with NSCLC,^[2]^ losing the opportunity for surgery, are already in advanced stages at the time of diagnosis. With the development of medicine, molecular-targeted therapy and immunotherapy have emerged, but chemotherapy is still the cornerstone of NSCLC.^[3]^ The combination of 2 drugs containing platinum is one of the classic chemotherapy regimens. With the enrichment of chemotherapy drugs, gemcitabine (GEM) is a new generation of pyrimidine antitumor drugs. GEM mainly acts in the DNA synthesis phase and G1 phase, which can effectively block the transition of cells from G1 to S phase, and plays a broad-spectrum antitumor role.^[4,5]^ It has gradually become one of the commonly used chemotherapy drugs in NSCLC. GEM alone or combined with platinum drugs has been widely used in the treatment of NSCLC.^[6,7]^ Related studies have shown that GEM combined with cisplatin (GP scheme) has obvious survival benefits compared with GEM alone or combined with other platinum drugs (HR = 0.9, 95% CI 0.84–0.96, *P* < .001).^[8]^

However, in the process of receiving GP, NSCLC patients may have gastrointestinal reactions, blood toxicity, bone marrow suppression, and other toxic side effects, which affects the quality of life of patients and shortens the survival time of patients. Therefore, in the treatment of NSCLC, ensuring the quality of life of patients is as important as prolonging the survival time of patients. At present, a large number of studies have proved that traditional Chinese medicine has advantages in inhibiting tumor growth, regulating immunity, synergizing with modern medical treatment, and so on.^[9–11]^ Chinese medicine injection (CHIs) is guided by the theoretical system of syndrome differentiation of traditional Chinese medicine, combined with the purification of modern advanced technology. It has the characteristics of rapid efficacy and convenient application. In clinical therapy, CHIs is a complementary or alternative therapy for GP regimen chemotherapy.

Therefore, this study intends to conduct a meta-analysis of randomized controlled trials of CHIs combined with GP scheme in the treatment of advanced NSCLC, to explore the potential rank of the efficacy and safety.

## 2 Method

### 2.1 Study registration

The protocol for this systematic review and network meta-analysis has been registered under PROSPERO platform with the registration number of CRD42020167142, and will be reported following the Preferred Reporting Item for Systematic Reviews and Meta-Analysis Protocols (PRISMA-P) checklist.

### 2.2 Inclusion criteria for study selection

#### 2.2.1 Types of studies

All the RCTs of CHIs plus GP chemotherapy compared with GP alone for advanced NSCLC will be included, and the languages will not be restricted. The outcome should include at least one of the objective response rate (ORR) or adverse reactions (nausea and vomiting, leukopenia). Beside, full journal publication with sufficient data for analysis will be required.

#### 2.2.2 Types of patients

The patients had NSCLC with stages III to IV diagnosed, conforming with histopathological and cytological diagnostic criteria, and TNM classification was based on American Joint Committee on Cancer.^[12]^ Patients did not receive any radiotherapy, other chemotherapy, or Chinese herbs during this study.

#### 2.2.3 Types of interventions

Intervention: the control group was given GP regimen chemotherapy, while the experimental group was additionally given CHIs on the basis of GP regimen chemotherapy. CHIs therapy interventions, including 11 CHIs for lung cancer^[5]^: Aidi injection (ADI), Toad Venom Injection (TVI), Compound Kushen injection (CKSI), Huachansu injection (HCSI), Kanglaite injection (KLTI), Kangai injection (KAI), Brucea javanica Oil Emulsion injection (BJOEI), Shenqi Fuzheng injection (SQFZI), Xiaoaiping injection (XAPI), Ginseng Polysacchride Injection (GPI), Astragalus polysaccharide injection (API).

#### 2.2.4 Types of outcome measures

##### 2.2.4.1 Primary outcomes

ORR according to the World Health Organization (WHO) guidelines for solid tumor responses ^[13]^ or Response Evaluation Criteria in Solid Tumors (RECIST),^[14]^ indicators were complete response (CR), partial response (PR), stable disease (SD), progressive disease (PD), ORR being equal to CR plus PR. Adverse reactions (ADEs or ADRs) were pooled, including nausea and vomiting, leukopenia.

##### 2.2.4.2 Secondary outcomes

The long-term synergistic efficacy of this combination was considered as MST. In addition, the secondary outcomes included disease control rate (DCR) and quality of life (QOL). QOL was considered improved if the KPS score increased by 10 points or higher after treatment.^[15]^ DCR is equal to CR plus PR and SD.

### 2.3 Data sources and search strategy

**Figure 1:**
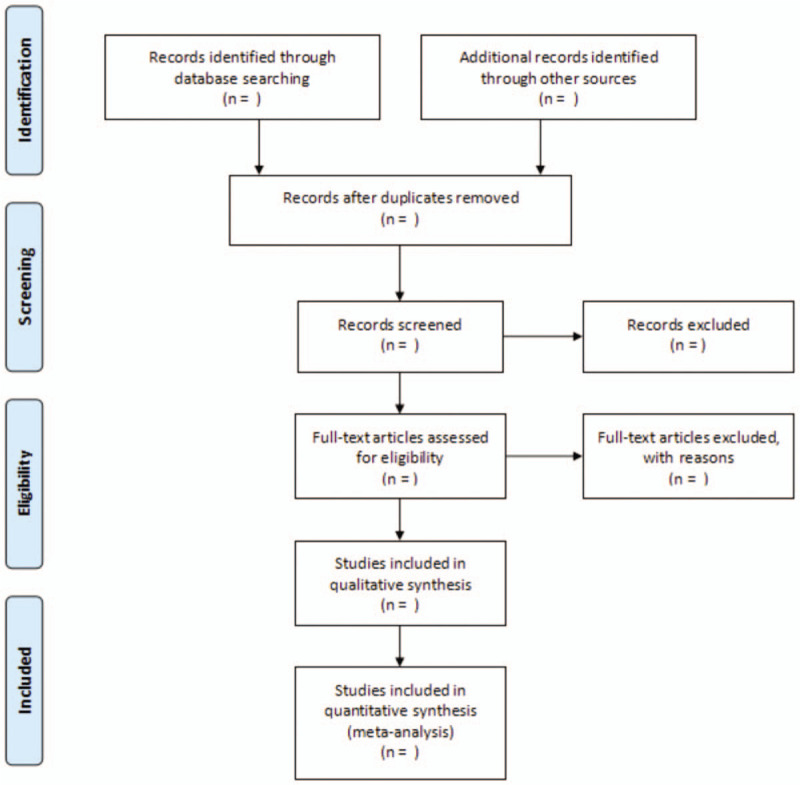
Flow diagram of studies search and selection. *From:* Moher D, Liberati A, Tetzlaff J, Altman DG, The PRISMA Group (2009). *P*referred *R*eporting *I*tems for *S*ystematic Reviews and *M*eta-*A*nalyses: The PRISMA Statement. PLoS Med 6(7): e1000097. doi:10.1371/journal.pmed100009.

Two authors (JL, G-HZ) will independently conduct a comprehensive systematic search of clinical trials published on electronic databases including PubMed, Embase, Web of Science (ISI), Cochrane Central Register of Controlled Trials (CENTRAL), China National Knowledge Infrastructure Database (CNKI), Chinese Scientific Journals Full-Text Database (VIP), Wanfang Database and China Biological Medicine Database (CBM) from inception to May 2020. All retrievals will be implemented using MeSH and free word. The combination of the following MeSH terms, “Nonsmall cell lung cancer,” “nonsmall lung carcinoma,” “NSCLC,” “gemcitabine,” “cisplatin,” and search terms for each CHI ^[16]^ will be used. Furthermore, any additional relevant articles were identified by searching studies that were included in the reference lists from existing systematic reviews and meta-analyses. All unclear questions were addressed by contacting the study authors by email. The selection process will be summarized according to the PRISMA flow diagram (Fig. 1).

### 2.4 Data collection and analysis

#### 2.4.1 Selection of studies and data extraction

Two researchers (G-HZ, B-WX) will screen titles and abstracts independently, after duplicate removal by one of the investigators. Consensus will be sought in case of initial disagreement. If consensus cannot be reached, the report will be included for full-text evaluation. Then, 2 researchers independently extracted the following information from each study: lead author, publication time, demographic characteristics, sample size, CHIs therapies, evaluation criteria of clinical efficacy, duration of follow-up, relevant indicators of bias risk assessment, and supportive treatment such as antinausea drugs, granulocyte colony-stimulating factor. Furthermore, outcomes including the ORR, leukopenia, nausea and vomiting, MST, DCR, and QOL will be examined. If above-mentioned information is not able to get, we will contact the corresponding author for detailed data. Any disagreements will be resolved by a third reviewer (JL).

#### 2.4.2 Risk of bias and reporting quality of included trials

The methodological quality of the included RCTs was assessed independently by 2 researchers (JL, T-TL) based on the Cochrane risk-of-bias criteria.^[17]^ Seven domains including selection bias, performance bias, detection bias, attrition bias, reporting bias, and other bias would be judged with low, unclear, and high risk. If there are divided opinions between 2 researchers in procession, we will resolve inconsistencies through discussion or asking for a help from a senior researcher.

#### 2.4.3 Anticipated structure of network

Network geometry will use nodes to represent GP plus different CHIs, and connected lines to represent the head-to-head comparisons between network nodes. The size of nodes and connected lines will represent the sample sizes of intervention and number of RCTs comparing such 2 treatment regimens. We will categorize the network nodes as follows: GP plus ADI, GP plus TVI, GP plus CKSI, GP plus HCSI, GP plus KLTI, GP plus KAI, GP plus BJOEI, GP plus SQFZI, GP plus XAPI, GP plus GPI, and GP plus API, subject to the condition they are compared in eligible RCTs.

#### 2.4.4 Data synthesis

We will model networks following the Bayesian approach, using WinBUGS 14 and Stata 15.1 software. Dichotomous data will be presented as odd ratio (OR) with 95% CI. For survival outcomes, MST will be presented as hazard ratio (HR) with 95% CI. The HR, if not reported, will be extracted from the survival curves and event rates by the method described by Parmar et al.^[18]^ Conventional meta-analyses will be conducted using a random effects model. Heterogeneity across head-to-head trials will be assessed using I^2^ statistics. The values of 25%, 50% and 75% for the I^2^ as indicative of low, moderate, and high statistical heterogeneity, respectively.

Pairwise meta-analysis will be conducted according to heterogeneity. A frequentist framework, random-effects network meta-analysis will be used to compare all classes of CHIs for each prespecified outcome. The surface under the cumulative ranking area (SUCRA) will be conducted to rank probabilities of interventions.

If sufficient data are available, we plan to conduct subgroup analyses to explore statistical heterogeneity across trials and inconsistency between direct and indirect evidence.

A comparison-adjusted funnel plot will be used to identify whether there will be a publication bias effect among the networks.

### 2.5 Evidence quality grating

Evidence quality will be conducted by the Graduates Assessments, Development and Evaluation (GRADE) established by the World Health Organization with the results of high quality, medium quality, low quality, and very low quality.

### 2.6 Patients and public participation

This is a systematic review and network meta-analysis, so it will not involve patient and public data.

### 2.7 Ethics and communication

Since this systematic review will not involve raw data collection, no ethical review is required. The authors will disseminate this systematic review through peer-review publications.

## 3 Discussion

The present systematic review will evaluate the comparative efficacy and safety of CHIs combined with GP regimen chemotherapy. Besides, the long-term synergistic efficacy of this combination will be concerned. We also review the quality of evidence using GRADE approach. Therefore, we hope the evidence from this systematic review could be useful for patients, clinical practitioners, and guideline-makers to select an optimum proposal of CHIs plus GP for advanced NSCLC.

## Author contributions

Conceptualization: Juan Li, Guang-Hui Zhu; Data curation: Tong-Tong Liu, Bo-Wen Xu; investigation: Bo-Wen Xu, Guang-Hui Zhu; Methodology: Juan Li, Guang-Hui Zhu, Tong-Tong Liu; Supervision: Jie Li; Writing – original draft: Juan Li, Guang-Hui Zhu; Writing – review & editing: Juan Li.
